# Clinical and genetic characteristics of adult patients with familial Mediterranean fever at a German tertiary referral centre

**DOI:** 10.1186/s41927-026-00684-2

**Published:** 2026-08-28

**Authors:** Dorothea Reck, Jörg Christoph Henes, Sebastian Jonas Saur

**Affiliations:** 1 Division of Internal Medicine, University Hospital Basel, Petersgraben 4, Basel, 4031 Switzerland; 2 Centre for Interdisciplinary Clinical Immunology, Rheumatology and Autoinflammatory Diseases, Department of Internal Medicine II (Oncology, Haematology, Immunology and Rheumatology), University Hospital Tuebingen, Otfried-Mueller-Strasse 10, 72076 Tuebingen, Germany

**Keywords:** Hereditary autoinflammatory diseases, Hereditary periodic fever syndromes, Familial mediterranean fever, Amyloidosis, MEFV mutation

## Abstract

**Objective:**

To characterise the clinical and genetic profile of adult familial Mediterranean fever (FMF) patients at a German tertiary referral centre and examine genotype–phenotype associations.

**Methods:**

In this prospective single-centre registry study (October 2019–March 2021), 95 adult FMF patients at University Hospital Tübingen were evaluated using structured questionnaires and medical record review. All patients had a pre-existing clinical diagnosis of FMF based on the Tel Hashomer and/or Eurofever/PRINTO criteria. Demographic data, clinical manifestations, treatment, and MEFV mutations were analysed. Patients were stratified by mutation status (homozygous M694V, heterozygous/compound-heterozygous M694V, other mutations). Statistical analyses included the Fisher–Freeman–Halton exact test and ANOVA. Variants were classified according to the INSAID consensus classification; R202Q was treated as a benign polymorphism. All subgroup comparisons were exploratory and were not corrected for multiple testing.

**Results:**

Median age was 36 years, with median symptom onset at 10 years. Most patients were of Turkish origin (74.7%), and 59.6% reported a family history of FMF. Common manifestations included abdominal pain (90.5%), arthralgia/arthritis (75.8%), fever (66.3%), and chest pain (52.6%). Colchicine was used in 96.8% of patients, while 38.3% received biologic therapy, a figure influenced by concurrent participation in a tocilizumab trial at our centre. MEFV analysis had been performed in 91 of the 95 patients (95.8%); among these, at least one MEFV variant was detected in 87 (95.6%), with M694V being the most frequent variant (61.5%). Homozygous M694V patients showed earlier disease onset (nominal *p* = 0.02) and higher rates of arthralgia/arthritis (nominal *p* = 0.035). Amyloidosis occurred in one patient with homozygous M694V.

**Conclusion:**

This cohort demonstrates typical FMF features with a predominance of the M694V mutation. Homozygosity for M694V is associated with earlier disease onset and increased musculoskeletal involvement. Subgroup findings are exploratory and require confirmation in independent cohorts.

**Supplementary Information:**

The online version contains supplementary material available at https://doi.org/10.1186/s41927-026-00684-2.

## Introduction

Familial Mediterranean fever (FMF) is the most common monogenic autoinflammatory disease and is characterised by recurrent episodes of fever and serosal inflammation, typically presenting with abdominal pain, chest pain, arthritis, or skin manifestations [1]. The disease most frequently affects populations originating from the Mediterranean region and the Middle East, including individuals of Turkish, Arab, Armenian, Jewish and Iranian (Persian) descent [2, 3]. FMF is caused by mutations in the MEFV gene, which encodes pyrin, a protein involved in the regulation of the innate immune response and inflammasome activation [4]. Colchicine remains the cornerstone of treatment in FMF, and its regular use prevents inflammatory attacks and suppresses chronic subclinical inflammation. In patients who do not respond to or are intolerant of colchicine, anti-IL-1 agents have emerged as effective therapeutic options [5]. The efficacy of tocilizumab, an IL-6 receptor antagonist, has also been investigated in clinical studies, where some therapeutic benefit has been reported [6].

More than 400 MEFV variants have been described [7], although a limited number of mutations account for the majority of genetically confirmed FMF cases. Among these, mutations such as M694V, M680I, V726A, E148Q, and M694I are most commonly reported [8]. The distribution of MEFV mutations varies across ethnic groups and geographic regions, and certain mutations, particularly M694V, have been associated with earlier disease onset and a more severe clinical phenotype in some cohorts [9]. Despite these observations, genotype–phenotype correlations in FMF remain incompletely understood. Factors beyond the MEFV genotype itself, including epigenetic regulation of pyrin expression, have been proposed as contributors to phenotypic variability [10].

In addition, diagnostic delay remains a challenge in FMF, particularly in populations living outside traditionally endemic regions [11]. Variability in clinical presentation and differences in genetic background may contribute to this delay. Therefore, detailed characterisation of clinical manifestations, mutation patterns, and their distribution across ethnic groups remains important for improving disease recognition and understanding genotype–phenotype associations.

The aim of the present cohort study was to characterise the clinical features, demographic characteristics, and MEFV gene mutation patterns of adult patients with FMF. We examined mutation distributions across ethnic groups and assessed associations between genotype (heterozygous, homozygous, or compound-heterozygous) and clinical manifestations, including age at onset, familial occurrence, and symptom patterns.

## Materials and methods

We performed a prospective, single-centre, non-interventional registry study. Patients were recruited from the Rheumatology Outpatient Clinic of the Department of Internal Medicine, University Hospital Tübingen, over an 18-month period (October 2019–March 2021). Eligible subjects were adults (male and female) aged ≥ 18 years with a preexisting diagnosis of familial Mediterranean fever (FMF). Only patients who gave written informed consent after detailed verbal and written information were included in the analysis.

### Diagnosis of FMF

All participants had a pre-existing clinical diagnosis of FMF, established by experienced rheumatologists at our interdisciplinary centre for autoinflammatory diseases. Diagnoses were made using the Tel Hashomer criteria [12] and, for more recently diagnosed patients, the Eurofever/PRINTO classification criteria [13], interpreted together with the MEFV genotype where available. Because the registry enrolled patients with an already established diagnosis, the criteria applied at the time of the original diagnosis were not systematically re-verified for the present analysis; this is acknowledged as a limitation.

### Data collection

Data were obtained from two sources: patient-completed questionnaires and the patients’ medical records. Each participant completed a structured questionnaire that was developed specifically for the present study and had not been published previously. The questionnaire was administered exclusively in German; no translated version was used for data collection. The English version provided as Additional file 1 was prepared for publication only and was not used with patients; accordingly, no forward–backward translation procedure was applicable. The instrument was designed for the Tübingen registry for FMF and other autoinflammatory diseases as a whole, which also comprises patients with cryopyrin-associated periodic syndromes (CAPS), tumour-necrosis-factor-receptor-associated periodic syndrome (TRAPS) and adult-onset Still’s disease. It therefore also covers items such as hearing loss and organomegaly, which are characteristic of those diagnoses rather than of FMF and are reported here as coexisting findings. Responses indicating ‘since early childhood’ or ‘childhood’ were classified as onset before 5 years of age.

A second section of the questionnaire (items ≥ 16), covering prior abdominal surgery, comorbidities in the patient and comorbidities in the family, was added after recruitment had already begun, because these items were identified as relevant only after an initial review of the accumulating registry data. Patients already enrolled were invited to complete this section at a subsequent clinic visit or by post. The study therefore combines prospectively and retrospectively collected elements, and the items in this section have reduced and variable sample sizes. Where questionnaire responses were missing, the corresponding information was retrieved from the medical records where available; for the surgical items this was possible for appendectomy only.

Additional clinical and genetic data were extracted from current discharge letters and outpatient records accessed via the hospital information system IS-H (SAP SE, Walldorf, Germany). Extracted items included: diagnosis at the time of data analysis, disease-defining mutation(s) if known, date of first diagnosis, further disease manifestations not captured by the questionnaire, and presence of amyloidosis. All collected data from questionnaires and medical records were entered into a central spreadsheet for analysis.

### Ethnicity classification

Ethnicity was self-reported by patients from a fixed list of categories provided in the questionnaire. The category rendered as “Caucasian” denoted self-identified European ancestry outside the Mediterranean FMF-endemic region and is used in this sense throughout. This categorisation is neither genetically defined nor externally validated; patients of Greek origin — who are both European and from an FMF-endemic region — were assigned to the “other” category on the basis of their own responses. All ethnicity-based analyses are therefore exploratory.

### MEFV analysis and variant classification

MEFV analyses were performed as part of routine clinical care prior to study inclusion, at different laboratories and over a period spanning more than two decades. Genotypes were extracted from the diagnosis sections of the clinical records. The laboratory, the analytical technique (targeted mutation panel, Sanger sequencing or next-generation sequencing), the commercial kit where applicable, and the extent of the region analysed were not uniformly documented in these records and can therefore not be reported. Variants outside the classical exon 2 and exon 10 hotspots, including variants in exon 3, were documented in individual patients, indicating that at least some patients underwent analysis extending beyond the common variant panel. This heterogeneity is a limitation: the reported variant frequencies are likely to underestimate the true frequency of rarer variants in patients tested with restricted panels.

MEFV variants were classified according to the consensus classification of the International Study Group for Systemic Autoinflammatory Diseases (INSAID) [14] as recorded in the Infevers registry (34; accessed 8 August 2026). M694V, M694I, M680I, V726A and R761H were regarded as pathogenic or likely pathogenic. E148Q, P369S, R408Q and A744S are currently classified as variants of uncertain significance. R202Q is classified as a benign polymorphism and was therefore not counted as a disease-associated variant. Because the diagnosis of FMF in this cohort was established clinically, patients carrying only R202Q remain valid FMF cases and were not excluded from the cohort.

### Denominators

Denominators vary between analyses and are stated explicitly for each result. The full cohort comprised 95 patients. Medication data were available for 94 patients. MEFV analysis had been performed in 91 patients, who constitute the denominator for all genotype-based analyses (Tables 2 and 4); the remaining four patients had never undergone genetic testing. Zygosity could be determined in 85 patients (Table 1). Among Turkish patients, 68 of 71 had undergone MEFV analysis. Appendectomy status was known for 56 of the 91 genotyped patients, and information on the remaining surgical procedures for 47.

### Data management and software

Questionnaire and extracted record data were compiled in Microsoft Excel (Version 16.54; Microsoft Corporation, Redmond, WA, USA). Statistical analyses were performed using IBM SPSS Statistics (Version 28.0.1.1; IBM Corp., Armonk, NY, USA).

### Statistical analysis

For comparison of categorical variables, we used the Fisher–Freeman–Halton exact test for contingency tables when expected cell counts were < 5 (two-sided exact significance). For comparisons of means between groups, analysis of variance (ANOVA) was employed. The nominal significance level was set at α = 0.05. No corrections for multiple testing were applied. Tables 3 and 4 comprise a large number of subgroup comparisons; all reported p values are therefore nominal and uncorrected, and all subgroup analyses should be regarded as exploratory and hypothesis-generating rather than confirmatory. Several subgroups are small — most notably the four patients in whom genetic testing detected no MEFV variant — so that statistical power was limited and only large effect sizes could have been detected; non-significant results should not be interpreted as evidence of absence of an association. No a priori sample size or power calculation was performed, since the study was designed as a descriptive analysis of all consecutively consenting registry patients rather than to test a pre-specified hypothesis.

## Results

A total of 95 patients, diagnosed with FMF, were included. The cohort comprised 49 female (51.6%) and 46 male (48.4%) participants. At study inclusion, the age of patients ranged from 18 to 66 years (median 36, mean 36.2 ± 11.6). Age at disease onset ranged from 0 to 48 years (median 10, mean 14.5 ± 12.5). At the time of initial diagnosis, patients’ age ranged from 0 to 64 years (median 21, mean 22.7 ± 15.2). Discrepancies were observed between patient-reported age at disease onset and age at first documented diagnosis. The median interval from symptom onset to diagnosis was 4.5 years (mean 7.8 ± 11.5).

### Ethnicity

The majority of FMF patients were of Turkish origin (*n* = 71, 74.7%). Self-reported European (non-Mediterranean) ancestry, denoted “Caucasian” throughout, was reported by nine patients (9.5%), and Arab origin by nine patients (9.5%). Additional ethnic backgrounds included Greek origin (*n* = 3) as well as individual patients reporting Chechen, Aramaic, and Ashkenazi Jewish origin.

### Family history and parental consanguinity

A positive family history of the same disease was reported by 59.6% of FMF patients. Overall, 24 FMF patients (25.3%) reported parental consanguinity. Among the 71 Turkish FMF patients, 22 (31%) reported parental consanguinity, while seven patients (9.9%) indicated that the consanguinity status was unknown. One FMF patient of Caucasian origin reported parental consanguinity; the parents of this patient were from Armenia. One FMF patient of Arab origin also reported parental consanguinity.

### Clinical manifestations

Among patients with FMF, abdominal pain was the most frequently reported symptom, affecting 86 patients (90.5%). Arthralgia and/or arthritis were reported by 72 patients (75.8%), and fever by 63 patients (66.3%). Chest pain occurred in 50 patients (52.6%). Rash was reported by 15 patients (15.8%). Amyloidosis was present in one patient (1.1%). Rare manifestations included pharyngitis, conjunctivitis, and lymphadenopathy, each reported by one patient (1.1%). Splenomegaly and/or hepatomegaly (14 patients, 14.9%) and hearing loss (four patients, 4.2%) are reported here as coexisting clinical findings and not as manifestations of FMF; these items were included because the questionnaire was designed for the registry as a whole (see Methods).

### Medication

Medication history was available for 94 patients with FMF. Two patients (2.1%) had not received pharmacological treatment at the time of study inclusion. Overall, 91 patients (96.8%) were treated with colchicine, which was sufficient as monotherapy in 51 patients (54.3%). In 40 patients (42.6%), colchicine was discontinued and therapy was switched to alternative treatments. Nineteen patients each (20.2%) had received canakinumab or anakinra, and 11 patients (11.7%) tocilizumab within the TOFFIFE study [6].

At the time of data collection, 36 patients (38.3%) had been treated with at least one of the biologic agents canakinumab, anakinra, or tocilizumab. Eight patients (8.5%) received canakinumab alone and eight (8.5%) anakinra alone, while seven patients (7.4%) were treated with tocilizumab monotherapy. Nine patients (9.6%) received both canakinumab and anakinra, two (2.1%) canakinumab and tocilizumab, and two (2.1%) anakinra and tocilizumab.

Methotrexate was used in six patients (6.4%). Five patients (5.3%) received at least one TNF inhibitor due to concomitant autoimmune disease, including Crohn’s disease (*n* = 1), seronegative rheumatoid arthritis (*n* = 1), HLA-B27–negative spondyloarthritis (*n* = 1), undifferentiated oligoarthritis (*n* = 1), and juvenile idiopathic arthritis (*n* = 1). Azathioprine was administered to three patients (3.2%): one with Crohn’s disease, one with suspected FMF–Behçet overlap syndrome, and one with undifferentiated oligoarthritis.

### Mutation analysis

MEFV analysis had been performed in 91 of the 95 FMF patients (95.8%); the remaining four patients had never undergone genetic testing and were excluded from all genotype-based analyses. Among the 91 tested patients, at least one MEFV variant was detected in 87 (95.6%), whereas no variant was identified in four (4.4%). All mutation frequencies below are expressed as proportions of the 91 tested patients. The M694V mutation was the most frequent variant, occurring alone (heterozygous or homozygous) in 26 patients (28.6%) and in combination with other mutations in an additional 30 patients, resulting in a total of 56 patients (61.5%) carrying M694V. The most frequent compound mutations included M694V/V726A (8 patients, 8.8%), followed by M694V/E148Q (7 patients, 7.7%) and M694V/M680I (6 patients, 6.6%).

At least one of the five most common MEFV mutations (V726A, M694V, M694I, M680I, E148Q) was present in 77 patients (84.6%). M680I was detected in 19 patients (20.9%), E148Q in 18 patients (19.8%), and V726A in 15 patients (16.5%). Three patients (3.3%) carried at least one M694I mutation. Less frequent variants included R202Q (7.7%), P369S (5.5%), and R761H or R408Q (3.3% each), while several additional variants were observed only once.

### R202Q and reclassification

Seven of the 91 genotyped patients (7.7%) carried R202Q. In five of these, R202Q co-occurred with M694V (heterozygous R202Q with heterozygous M694V, *n* = 1; heterozygous R202Q with homozygous M694V, *n* = 1; homozygous R202Q with heterozygous M694V, *n* = 1; homozygous R202Q with homozygous M694V, *n* = 2). In the remaining two patients, R202Q was not accompanied by any pathogenic or likely pathogenic variant: one carried heterozygous R202Q alone, and one carried homozygous R202Q together with heterozygous P369S and heterozygous R197Q, both variants of uncertain significance. When R202Q is not counted as a disease-associated variant, the number of patients carrying at least one MEFV variant other than R202Q is 85 of the 91 tested patients (93.4%). Both reclassified patients belong to the other/other group in Table 4. In a sensitivity analysis excluding them from that group (*n* = 32 to *n* = 30), the association between mutation group and arthralgia/arthritis remained significant at the nominal level (*p* < 0.05), and no other result changed materially.

MEFV zygosity information was available for 85 patients (89.5%) (Table 1). Compound heterozygous mutations were the most frequent genotype (38.8%), followed by homozygous (29.4%) and heterozygous mutations (25.9%), while five patients (5.9%) carried both homozygous and heterozygous variants. Among homozygous mutations, M694V predominated (84%). Among heterozygous variants, M694V (31.8%) and M680I (27.3%) were most common. In compound heterozygous patients, the most frequent combinations were M694V/V726A (24.2%), M694V/E148Q (18.2%), and M694V/M680I (15.2%).

**Table 1:** Zygosity of MEFV mutations among FMF patients (*n* = 85). Absolute numbers and percentages of patients are shown

	FMF patients
Homozygous	25(29.4%)
M694V ᵃ	21 (84%)
M680I	2 (8%)
Other	2 (8%)
Heterozygous	22 (25.9%)
M694V	7 (31.8%)
M680I	6 (27.3%)
E148Q	3 (13.6%)
Other	6 (27.3%)
Compound-heterozygous	33 (38.8%)
M694V/V726A	8 (24.2%)
M694V/M680I	5 (15.2%)
M694V/E148Q	6 (18.2%)
M694V/R202Q	2 (6.1%)
M680I/V726A	2 (6.1%)
Other	10 (30.3%)
Homozygous and heterozygous ᵇ	5 (5.9%)

ᵃ Two of these 21 patients also carry homozygous R202Q; because R202Q is classified as a benign polymorphism (see Methods), they are reported here as homozygous M694V. In the previous version, these two patients were listed in a separate row labelled “M694V + R202Q”. ᵇ This category includes one patient who is homozygous for M694V and heterozygous for R202Q. Together with the 21 patients above, this gives the 22 patients in the M694V/M694V group of Table 4

### MEFV mutations according to ethnicity

The distribution of MEFV mutations varied across ethnic groups (Table 2). Among Turkish patients, M694V was the most frequent mutation, occurring in 20 patients (29.4%) in homozygous form and in 24 patients (35.3%) in heterozygous or compound heterozygous form. The second most common mutation was M680I (2.9% homozygous, 19.1% heterozygous or compound heterozygous), followed by E148Q (17.6%). V726A was detected in 14.7% of Turkish patients and R202Q in 7.4%.

Among Caucasian patients, M694V was also the most frequent mutation (44.4%), followed by E148Q (33.3%). Individual patients carried heterozygous or compound heterozygous M680I or M694I mutations, or a homozygous R202Q mutation.

**Fig. 1:**
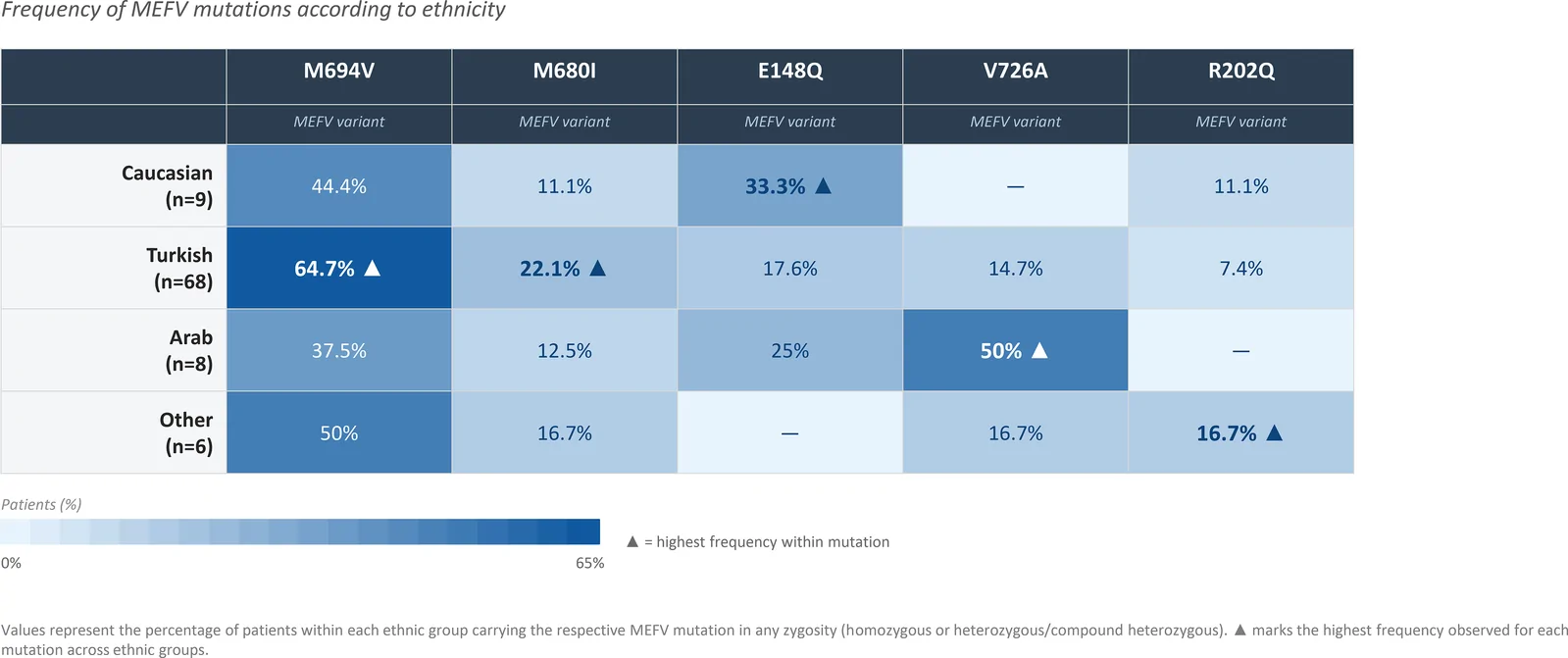
Frequency of MEFV mutations according to ethnicity. The five most common variants (M694V, M680I, E148Q, V726A, R202Q) are shown as proportions of all genotyped patients within each ethnic group (Caucasian *n* = 9, Turkish *n* = 68, Arab *n* = 8, other *n* = 6). Each cell shows the combined frequency of homozygous and heterozygous/compound heterozygous occurrences within that group; a dash denotes a frequency of zero. Colour intensity encodes the same percentage and carries no additional information. R202Q is classified as a benign polymorphism (see Methods) and is shown for completeness only; it should not be interpreted as a disease-associated variant. Owing to the small size of the non-Turkish subgroups, between-group differences in this figure are exploratory

Among Arab patients, V726A was the most frequent mutation (50%), including one homozygous case, followed by M694V (37.5%). E148Q and M694I were each observed in two patients (25%). Among patients of other ethnicities, M694V was present in three patients (50%), while individual cases carried M680I, V726A, or R202Q mutations. The distribution of key mutations across ethnic groups is visualised in Fig. 1. Clinical manifestations stratified by ethnicity are presented in Table 3. All nine Caucasian patients reported arthralgia or arthritis (100%), compared with 76.1% of Turkish, 44.4% of Arab, and 83.3% of patients of other ethnicity; the difference between Caucasian and Arab patients reached nominal significance (uncorrected *p* = 0.029).

**Table 2:** MEFV mutations of FMF patients according to ethnicity, calculated among the 91 patients in whom MEFV analysis had been performed; the four patients who never underwent genetic testing are excluded

Genotype	Caucasian (*n* = 9)	Turkish (*n* = 68)	Arab (*n* = 8)	Other (*n* = 6)
M694V/M694V	1 (11.1%)	20 (29.4%)	0	1 (16.7%)
M694V/*	3 (33.3%)	24 (35.3%)	3 (37.5%)	2 (33.3%)
M694I/*	1 (11.1%)	0	2 (25%)	0
M680I/M680I	0	2 (2.9%)	1 (12.5%)	0
M680I/*	1 (11.1%)	13 (19.1%)	0	1 (16.7%)
V726A/V726A	0	1 (1.5%)	1 (12.5%)	0
V726A/*	0	9 (13.2%)	3 (37.5%)	1 (16.7%)
E148Q/E148Q	0	0	1 (12.5%)	0
E148Q/*	3 (33.3%)	12 (17.6%)	1 (12.5%)	0
R202Q/R202Q	1 (11.1%)	3 (4.4%)	0	0
R202Q/*	0	2 (2.9%)	0	1 (16.7%)
Other	2 (22.2%)	16 (23.5%)	0	0

Data are absolute numbers and percentages within each ethnic group. * = no mutation at this locus or another mutation, including heterozygous and compound heterozygous variants. Note that R202Q is classified as a benign polymorphism (see Methods) and is shown for completeness only

**Table 3:** Clinical manifestations of FMF patients according to ethnicity. Data are presented as absolute numbers and percentages within each ethnic group. *p = 0.029 for arthralgia/arthritis between Caucasian and Arab patients (Fisher–Freeman–Halton exact test); this p value is nominal and uncorrected for multiple testing. Splenomegaly/hepatomegaly and hearing loss are reported as coexisting clinical findings, not as manifestations of FMF

Symptom	Caucasian (*n* = 9)	Turkish (*n* = 71)	Arab (*n* = 9)	Other (*n* = 6)
Fever	7 (77.8%)	45 (63.4%)	6 (66.7%)	5 (83.3%)
Abdominal pain	7 (77.8%)	64 (90.1%)	9 (100%)	6 (100%)
Chest pain	7 (77.8%)	37 (52.1%)	4 (44.4%)	2 (33.3%)
Arthralgia/Arthritis	9 (100%)*	54 (76.1%)	4 (44.4%)*	5 (83.3%)
Rash	1 (11.1%)	12 (16.9%)	1 (11.1%)	1 (16.7%)
Splenomegaly/Hepatomegaly ᶜ	2 (22.2%)	8 (11.3%)	3 (33.3%)	1 (16.7%)
Hearing loss ᶜ	0	4 (5.6%)	0	0

ᶜ Coexisting clinical finding, not regarded as a manifestation of FMF

Clinical manifestations of FMF patients stratified by mutation group: FMF patients in whom MEFV analysis had been performed (*n* = 91) were stratified into four groups based on their MEFV mutations (Table 4): homozygous M694V, heterozygous or compound-heterozygous M694V, other mutations, and no detectable variant. Patients homozygous for M694V exhibited symptom onset significantly earlier than the other groups (*p* = 0.02), and diagnosis was also made significantly earlier in this group (*p* = 0.002). A positive family history was more frequent among homozygous M694V patients (72.7%) compared with the other groups (54.5%, 53.1%, and 50%). Nearly all homozygous M694V patients (90.9%) were of Turkish descent. Accordingly, a homozygous M694V mutation was present in 29.4% of Turkish patients, 11.1% of Caucasian patients, and none of the Arab patients. Heterozygous or compound-heterozygous M694V mutations were observed in 35.3% of Turkish, 33.3% of Caucasian, and 37.5% of Arab patients. Other mutations without M694V occurred in 33.8% of Turkish, 33.3% of Caucasian, and 50% of Arab patients.

Regarding clinical manifestations, a nominally significant difference was observed among the four mutation groups for the occurrence of arthralgia and arthritis (uncorrected *p* = 0.035): 95.5% of homozygous M694V patients reported these symptoms, compared with 63.6% of heterozygous or compound-heterozygous patients and 71.9% of patients with other mutations. Fever was reported in 81.8% of homozygous M694V patients, compared with 57.6% (heterozygous/compound-heterozygous M694V) and 62.5% (other mutations). Chest pain was present in 72.7%, 48.5% and 43.8% of the three groups, respectively. Appendectomy had been performed in 69.2% of homozygous M694V patients, compared with 52.4% and 36.8% in the other two groups. These differences were not significant; full values for all variables are given in Table 4 and are not repeated here. Amyloidosis was observed in one FMF patient, who carried a homozygous M694V mutation.

**Table 4:** FMF patients stratified by mutation group: homozygous M694V (M694V/M694V), heterozygous or compound heterozygous M694V (M694V/other), mutations without M694V (other/other), and no detectable MEFV variant (none). The four patients who never underwent genetic testing are not included; the columns therefore sum to 91. Absolute numbers and percentages within each mutation group are shown. Mean comparisons were performed using ANOVA, and categorical variables were compared with the Fisher–Freeman–Halton exact test. All p values are nominal and uncorrected for multiple testing. n.s. = not significant

	M694V/M694V (*n* = 22)	M694V/other (*n* = 33)	other/other (*n* = 32)	none (*n* = 4)	Significance
Age at symptom onset (years)	7.7 ± 10.1	16.1 ± 11.5	18.9 ± 14.2	16 ± 11.2	0.02*
Age at diagnosis (years)	12.6 ± 14.5	27.7 ± 15.7	25.6 ± 13.6	24 ± 8.3	0.002*
Delay of diagnosis (years)	5.3 ± 12.7	11.2 ± 14.5	6.8 ± 7.6	8 ± 6.1	n.s.
Positive family history of FMF	16 (72.7%)	18 (54.5%)	17 (53.1%)	2 (50%)	n.s.
Parental consanguinity	6 (27.3%)	7 (21.2%)	10 (31.3%)	0	n.s.
Patient comorbidity	6 (27.3%)	3 (9.1%)	9 (28.1%)	1 (25%)	n.s.
Family comorbidity	6 (27.3%)	8 (24.2%)	5 (15.6%)	1 (25%)	n.s.
Ethnicity					
Caucasian	1 (4.5%)	3 (9.1%)	3 (9.4%)	2 (50%)	n.s.
Turkish	20 (90.9%)	24 (72.7%)	23 (71.9%)	1 (25%)	n.s.
Arab	0	3 (9.1%)	4 (12.5%)	1 (25%)	n.s.
Other	1 (4.5%)	3 (9.1%)	2 (6.3%)	0	n.s.
Symptoms					
Fever	18 (81.8%)	19 (57.6%)	20 (62.5%)	2 (50%)	n.s.
Abdominal pain	21 (95.5%)	31 (93.9%)	27 (84.4%)	3 (75%)	n.s.
Chest pain	16 (72.7%)	16 (48.5%)	14 (43.8%)	2 (50%)	n.s.
Rash	6 (27.3%)	3 (9.1%)	5 (15.6%)	0	n.s.
Hearing loss ᶜ	1 (4.5%)	0	3 (9.4%)	0	n.s.
Arthralgia/Arthritis	21 (95.5%)	21 (63.6%)	23 (71.9%)	3 (75%)	0.035*
Pharyngitis	0	0	1 (3.1%)	0	n.s.
Spleno-/Hepatomegaly ᶜ	4 (18.2%)	5 (15.2%)	4 (12.5%)	1 (25%)	n.s.
Conjunctivitis	0	0	1 (3.1%)	0	n.s.
Lymphadenopathy	0	0	1 (3.1%)	0	n.s.
Amyloidosis	1 (4.5%)	0	0	0	n.s.
Prior surgical procedures					
Appendectomy ᵃ	9 (69.2%)	11 (52.4%)	7 (36.8%)	0	n.s.
Other intestinal segments ᵇ	3 (30%)	1 (5.9%)	2 (11.8%)	0	n.s.
Cholecystectomy ᵇ	1 (10%)	1 (5.9%)	1 (5.9%)	0	n.s.
Oophorectomy ᵇ	2 (20%)	1 (5.9%)	1 (5.9%)	0	n.s.
Splenectomy ᵇ	0	0	1 (5.9%)	0	n.s.
Laparoscopy ᵇ	2 (20%)	5 (29.4%)	1 (5.9%)	0	n.s.

ᵃ Appendectomy status was known for 56 of the 91 genotyped patients (M694V/M694V *n* = 13, M694V/other *n* = 21, other/other *n* = 19, none *n* = 3). For this item, questionnaire responses were supplemented by information retrieved from the medical records, which was available for appendectomy only. ᵇ For all other surgical procedures, information was available from the questionnaire alone, in 47 of the 91 genotyped patients (*n* = 10, *n* = 17, *n* = 17 and *n* = 3, respectively). The two subsets are nested: every patient included in the smaller subset is also included in the larger one. ᶜ Coexisting clinical finding, not regarded as a manifestation of FMF

## Discussion

The present study provides a comprehensive characterization of a cohort of 95 patients with FMF at a German tertiary referral centre. Our findings emphasise the impact of the MEFV genotype on the clinical phenotype and highlight the challenges regarding diagnostic delay. All subgroup comparisons reported below are exploratory and uncorrected for multiple testing.

### Genotype–phenotype correlation and the role of M694V

The most frequent mutation in our FMF cohort was M694V, carried by 61.5% of genotyped patients. This finding is consistent with previous reports showing that M694V is the most prevalent mutation among FMF patients worldwide. In comparable studies, the M694V mutation has been reported in 33–66% of FMF patients, consistently representing the most common variant [15–19]. Analysis of mutation frequencies by ethnicity demonstrated that the M694V mutation was most prevalent among Turkish and Caucasian patients, whereas the V726A mutation predominated in Arab patients. These findings are consistent with existing literature, which reports M694V as the most frequent mutation in Turkish populations and V726A as the most common among Arab patients [20]. In a cohort of 500 Egyptian paediatric FMF patients, the M680I, M694V and V726A genotypes were directly compared, and genotype was associated with both attack pattern and disease severity [21].

One of the most striking findings of this study is the association between the homozygous M694V mutation and a more severe disease phenotype. Patients in this group exhibited a significantly earlier symptom onset (*p* = 0.02) compared to those with other mutations. Furthermore, arthralgia and arthritis were nearly universal in the homozygous M694V group (95.5%) compared to other mutation groups (nominal *p* = 0.035, uncorrected). These results align with previous research indicating that specific MEFV variants, particularly M694V, serve as major predictors of disease severity and specific organ involvement [22–26]. A recent study specifically characterising arthritis patterns in paediatric FMF according to exon 10 biallelic pathogenic genotypes similarly identified genotype-dependent differences in joint involvement [27].

Our observation that both early symptom onset and a positive family history were more frequent among homozygous M694V patients is mirrored in a recent nationwide Turkish study of 3,981 paediatric FMF patients, in which early onset and a positive family history were independent predictors of the homozygous M694V genotype and were incorporated into a clinical scoring system [28]. Our data suggest that this relationship also holds in an adult cohort recruited outside the endemic region, although the direction of inference in our cross-sectional design is descriptive rather than predictive.

Overall, abdominal pain was the most common symptom, reported by over 90% of patients. This high prevalence likely explains the frequent history of surgical interventions, particularly appendectomies, which had been performed in 69.2% of homozygous M694V patients. It has been demonstrated that appendicitis represents the most frequent misdiagnosis among FMF patients who are diagnosed at a later stage; that study reported that 84% of all FMF patients initially received an incorrect diagnosis [29]. These findings suggest that FMF attacks are still frequently misidentified as acute surgical abdomens, leading to unnecessary invasive procedures.

### Amyloidosis

Amyloidosis was observed in a single patient (1.1%), who carried a homozygous M694V mutation. Reported amyloidosis rates in FMF cohorts vary widely, from 0 to 13% [18, 19, 30–32]. It has been suggested that amyloidosis is less frequent among FMF patients who have migrated abroad than among those still living in their country of origin, and that better access to colchicine contributes to this difference [20, 33, 34]. With a single affected patient, however, our data cannot support any inference about the determinants of amyloidosis risk; this explanation is speculative and is not directly supported by the data presented here. For the same reason, the fact that this patient carried a homozygous M694V genotype is consistent with, but cannot corroborate, previous reports of a higher amyloidosis risk in this genotype [19, 24, 35], an association that other studies have not confirmed [36, 37].

### Biologic therapy

Overall, 38.3% of our patients had received at least one biologic agent, which is high compared with most published FMF cohorts. Several factors account for this. Eleven patients (11.7%) received tocilizumab within the randomised, double-blind, placebo-controlled TOFFIFE trial conducted at our centre during the recruitment period [6], and for seven of these patients tocilizumab was the only biologic agent received; excluding these seven, the proportion treated with a biologic agent falls to 30.9%. Beyond trial participation, our centre is a tertiary interdisciplinary referral centre for autoinflammatory diseases, to which patients with colchicine resistance or intolerance are preferentially referred; colchicine was discontinued and treatment switched in 42.6% of our patients. Access to anti-IL-1 agents and to clinical trials is also comparatively good in Germany, whereas cost and limited availability constrain their use in several of the countries from which comparator cohorts have been reported. The high rate of biologic treatment should therefore be interpreted as a consequence of referral bias and trial participation rather than as representative of unselected FMF patients in Germany.

### Diagnostic delay

Despite advances in genetic testing, our data reveal a substantial mean diagnostic delay of 7.8 years. These values are within the range reported in Turkish cohort studies, which describe delays between 8.8 and 11.2 years [16, 19, 31]. In a Dutch cohort, the mean time to diagnosis was 8.2 ± 3 years after disease onset [15], while a Greek cohort reported a delay of 11.4 years [38]. A Turkish study that also included paediatric patients demonstrated a slightly shorter delay of 6.9 ± 7.7 years [30]. This delay is likely attributable to overlapping clinical features with other disorders, variable disease courses, and a lack of awareness among physicians outside of specialised centres. Even in Turkey, where FMF prevalence is higher, one study reported a diagnostic delay of 11 years [29]; that study also demonstrated that the delay was significantly shorter in patients diagnosed after the year 2000 compared to those diagnosed earlier (15 vs. 4 years). Similarly encouraging trends have been observed in a German paediatric FMF cohort published in 2013, in which the mean interval to diagnosis was only 1.9 years [39]. These developments suggest that the delay between symptom onset and diagnosis may continue to decrease. Increased awareness of FMF, particularly with regard to its higher prevalence in certain ethnic groups, could play a key role in facilitating earlier diagnosis and improving patient care.

Interestingly, patients with homozygous M694V mutations were diagnosed significantly earlier (*p* = 0.002). This association has also been confirmed in previous studies, which demonstrated that patients with homozygous M694V mutations both develop symptoms at a younger age and are diagnosed earlier [24, 25]. Homozygous carriage of the M694V mutation therefore appears to be associated with earlier disease onset and earlier diagnosis.

### Ethnic diversity and consanguinity

While the majority of our cohort was of Turkish origin (74.7%), the presence of Caucasian (9.5%) and Arab (9.5%) patients underlines the increasing ethnic diversity of FMF in Germany and reflects that FMF should not be excluded on the basis of non-Mediterranean ancestry alone. Ethnic background was associated with differences in clinical presentation. Notably, all nine Caucasian patients reported arthralgia or arthritis (100%), a higher rate compared with Arab patients (44.4%; nominal *p* = 0.029), as shown in Table 3. Chest pain was more common in Caucasian patients (77.8%) compared with Turkish (52.1%) and Arab patients (44.4%), while abdominal pain was reported by all Arab patients (100%) and all patients of other ethnicity (100%).

These ethnicity-based comparisons must be regarded as exploratory. Outside the Turkish group the subgroups are very small (nine patients of self-reported European ancestry, nine Arab patients and six patients of other ethnicity), the categories were self-reported from a fixed list and are not genetically defined, and no correction for multiple testing was applied. A difference based on nine patients per group is highly sensitive to the classification of individual patients. These observations are hypothesis-generating and require confirmation in larger, prospectively defined cohorts before any clinical inference is drawn.

We observed a notably high rate of parental consanguinity (25.3% overall; 31.0% in the Turkish subgroup). This is consistent with high consanguinity rates reported in Turkish populations and may contribute to the elevated frequency of homozygous M694V mutations in this subgroup [20, 33]. The higher prevalence of homozygous genotypes in consanguineous families carries clinically relevant implications: homozygous M694V patients in our cohort had an earlier disease onset and more frequent musculoskeletal involvement. Consanguinity should therefore be enquired about routinely as part of the family history in patients with suspected autoinflammatory disease, particularly in patients of Turkish or Middle Eastern origin, and it may guide genetic counselling.

### Limitations

This is a single-centre study at a tertiary German referral centre; thus, the results may not be generalisable to all FMF patients. Referral bias towards more severe or atypical cases is likely, and is reflected in the high rate of biologic treatment discussed above. Data collection was partially retrospective and relied on patient-completed questionnaires, which may introduce recall bias. Additionally, the introduction of a second part of the questionnaire during the study led to reduced sample sizes for certain clinical items. Finally, we did not use validated severity scores (Pras or ISSF), which could have standardised comparisons.

No adjustment for multiple testing was performed. Given the number of subgroup comparisons in Tables 3 and 4, the two nominally significant findings (arthralgia/arthritis by mutation group, *p* = 0.035; arthralgia/arthritis in Caucasian versus Arab patients, *p* = 0.029) may represent chance findings and require confirmation in independent cohorts; they should not be regarded as confirmatory evidence. No a priori sample size or power calculation was performed, and several subgroups were small, so that only large effects could have been detected.

Genetic testing was not standardised. MEFV analyses were performed in routine care at different laboratories over more than two decades, and neither the technique nor the extent of the region analysed could be reconstructed from the records. Reported frequencies of rarer variants are therefore likely to be underestimates. In addition, several variants observed in our cohort — including E148Q, P369S and R408Q — are currently classified as variants of uncertain significance, so that the proportion of patients with a genetically confirmed diagnosis under strict INSAID criteria is lower than the proportion in whom any MEFV variant was detected.

Diagnoses were established clinically before study inclusion and were not systematically re-verified against a single set of criteria for this analysis. Skin involvement was captured only as unspecified “rash”; erysipelas-like erythema, the characteristic cutaneous manifestation of FMF, was not recorded separately, and the reported rash frequency of 15.8% can therefore not be attributed to FMF-specific skin disease. The ethnicity categories were self-reported and are neither genetically defined nor externally validated. The questionnaire was administered in German only; as the majority of our patients were of Turkish origin and German was for many not their first language, comprehension of individual items cannot be guaranteed. Finally, with a single patient affected by amyloidosis, no conclusions regarding amyloidosis risk can be drawn from these data.

## Conclusion

In conclusion, the MEFV genotype remains a critical determinant of the FMF clinical course. The homozygous M694V mutation is associated with earlier onset and higher rates of joint involvement, although this association was observed in exploratory analyses and requires confirmation. Reducing the persistent diagnostic delay remains a priority, requiring increased clinical suspicion and early referral to specialised centres, particularly in ethnically diverse populations.

## Supplementary Information

Below is the link to the electronic supplementary material.


**Supplementary Material 1:** Additional file 1. Supplementary questionnaire (English translation, for publication only; the instrument was administered in German).


## Data Availability

The datasets generated and analysed during this study are not publicly available because they contain potentially identifying clinical and genetic patient data (GDPR; ethics approval 505/2019BO2), but are available from the corresponding author on reasonable request. The study questionnaire is provided as Supplementary Information.
