# Supplementary material for: Clinical and genetic characteristics of adult patients with familial Mediterranean fever at a German tertiary referral centre
Source: BMC Rheumatol. 2026 Aug 28;10:65. doi: 10.1186/s41927-026-00684-2 (PMC13523296; doi:10.1186/s41927-026-00684-2)
Supplement: Supplementary file 1 [file 41927_2026_684_MOESM1_ESM.docx]

**Additional file 1**

**Supplementary questionnaire (English translation)**

*This structured questionnaire was developed specifically for the present study and had not been published previously. The original was administered in German; an English translation is provided here.*

These questions concern general information about you, your habits, and your disease. Please answer as truthfully as possible. Your answers will be treated confidentially.

**1.** How old were you when your disease first manifested?

**2.** What is your country of origin?

**3.** What is your father's country of origin?

**4.** What is your mother's country of origin?

**5.** Which ethnic group do you identify with?

• Caucasian (fair-skinned European)

• Turkish

• Arab

• African

• Asian

• Indian

• Indigenous

• Other: ____

**6.** Are your parents consanguineous (blood relatives)?

• Yes

• No

• Unknown

**7.** How many siblings do you have? ____

**8.** Are any other family members affected by your disease?

• No, I am the only one

• Yes: Mother / Father / Siblings (how many? ____) / Cousin / Aunt or Uncle

**9.** Do you have children? Yes (how many? ____; affected as well? ____) / No

**10.** Which religion do you identify with?

• Christian

• Muslim

• Buddhist

• Hindu

• Atheist

• Other: ____

**11.** Which option best reflects your highest level of education?

• I never attended school or left without a certificate

• Lower/intermediate secondary school or completed vocational training

• General or subject-specific higher-education entrance qualification (Abitur / Fachhochschulreife)

• Degree from a university of applied sciences, technician school, or master-craftsman school

• University degree (Magister, Diploma, State Examination) or higher

**12.** Do you smoke?

• Yes

• No

• I used to smoke

**13.** How does your disease typically manifest during flares? (tick all that apply)

• Fever

• Abdominal pain

• Chest pain

• Skin rashes

• Hearing impairment

• Joint pain / joint inflammation

• Other: ____

**14.** Are you diagnosed with any other autoimmune diseases? No / Yes:

• Autoimmune hepatitis

• Primary biliary cirrhosis

• Primary sclerosing cholangitis

• Hashimoto's thyroiditis

• Graves' disease

• Multiple sclerosis

• Myasthenia gravis

• Rheumatoid arthritis

• Psoriasis

• Crohn's disease

• Ulcerative colitis

• Malignancies (which: ____)

• Other: ____

**15.** Are any autoimmune diseases known in your closest relatives (father, mother, siblings, children)? If yes, in whom?

No / Yes — for each condition, specify Mother / Father / Sibling / Child: autoimmune hepatitis; primary biliary cirrhosis; primary sclerosing cholangitis; Hashimoto’s thyroiditis; Graves’ disease; multiple sclerosis; myasthenia gravis; rheumatoid arthritis; psoriasis; ulcerative colitis; Crohn’s disease; malignancies (which: ____); other: ____.

**16.** Have you ever undergone abdominal surgery? No / Yes — if yes, which type of operation?

• Appendectomy (removal of the appendix)

• Resection of other intestinal segments

• Cholecystectomy (removal of the gallbladder)

• Removal of ovarian cysts

• Splenectomy (removal of the spleen)

• Exploratory laparoscopy

• Gastric surgery

• Other operation: ____

**17.** Are there any foods that improve your disease or its symptoms? No / Yes — which? ____

**18.** Are there any foods that worsen your disease or its symptoms? No / Yes — which? ____

**19.** What do you believe is the cause of your disease? Why do you think you developed it?

• Fate

• Will of God

• Chance

• Consanguinity of my parents

• Environmental factors

• Other reason: ____

• I do not know
